# A Prediction Model for the Calculation of Effective Stiffness Ratios of Reinforced Concrete Columns

**DOI:** 10.3390/ma14071792

**Published:** 2021-04-05

**Authors:** Sourav Das, Iman Mansouri, Satyabrata Choudhury, Amir H. Gandomi, Jong Wan Hu

**Affiliations:** 1 Department of Civil Engineering, National Institute of Technology Silchar, Assam 788010, India; srv.das1@gmail.com (S.D.); scnitsilchar@gmail.com (S.C.); 2 Department of Civil Engineering, Birjand University of Technology, Birjand 97175-569, Iran; mansouri@birjandut.ac.ir; 3 Faculty of Engineering & IT, University of Technology Sydney, Ultimo 2007, Australia; amirhossein.gandomi@uts.edu.au; 4 Department of Civil and Environmental Engineering, Incheon National University, Incheon 22012, Korea; 5 Incheon Disaster Prevention Research Center, Incheon National University, Incheon 22012, Korea

**Keywords:** effective stiffness ratio, reinforced concrete columns, performance-based design, experimental data, gene expression programming, unified performance-based design

## Abstract

Nonlinear dynamic analyses of reinforced concrete (RC) frame buildings require the use of effective stiffness of members to capture the effect of cracked section stiffness. In the design codes and practices, the effective stiffness of RC sections is given as an empirical fraction of the gross stiffness. However, a more precise estimation of the effective stiffness is important as it affects the distribution of forces and various demands and response parameters in nonlinear dynamic analyses. In this study, an evolutionary computation method called gene expression programming (GEP) was used to predict the effective stiffness ratios of RC columns. Constitutive relationships were obtained by correlating the effective stiffness ratio with the four mechanical and geometrical parameters. The model was developed using a database of 226 samples of nonlinear dynamic analysis results collected from another study by the author. Subsequent parametric and sensitivity analyses were performed and the trends of the results were confirmed. The results indicate that the GEP model provides precise estimations of the effective stiffness ratios of the RC frames.

## 1. Introduction

The effective stiffness of reinforced concrete (RC) frame members significantly influences the computed nonlinear dynamic response of the structure. Improper estimation of member stiffness leads to an inaccurate evaluation of structural response quantities. Some of the important factors that are affected by member stiffness are the period of the structure, deformation demands, distribution of loads, and yield displacement [1,2,3,4,5,6]. Tensile strain developed in the section reduces the effective area of the RC section, and it is called a cracked section. Effective stiffness of a cracked section is less than that of an original uncracked section. As the neutral axis location is influenced by the steel quantity and distribution, the effective stiffness is a function of the strength of the section. Thus, to accurately estimate the response of RC buildings in nonlinear analyses, the use of effective stiffness based on strength is recommended. 

Generally, design codes recommend effective stiffness values as a fraction of gross stiffness, based on the axial load ratio. The American Society of Civil Engineers (ASCE 41-13) [7] have recommended flexural stiffness ratios from 0.3 to 0.7 for various reinforced concrete components. According to the Federal Emergency Management Agency (FEMA-356) [8], effective beam and column stiffness values of 0.5 and 0.5–0.7, respectively, are suggested based on the axial load ratio. However, from a previous study [9], it can be observed that these values do not accurately represent the response of the structure in the case of nonlinear dynamic analyses. Some researchers [10,11] have developed simplified effective stiffness models based on the axial load ratio. Kumar and Singh [11] also studied the effect of different parameters on the effective stiffness of RC sections. Kuntia and Ghosh [12] derived an expression for the effective stiffness of concrete columns that involves the axial load ratio, reinforcement, and eccentricity ratios, whereas Elwood and Eberhard [13] proposed a stiffness model that considers deformation due to bar slip, flexure, and shear. Comparative studies on the response of a building using different stiffness models were performed by Pique and Burgos [14], and the Priestley [1] model for structural response analysis of existing buildings was recommended. Similarly, Micelli et al. [15] studied the influence of various factors on the reduced stiffness and compared the behavior of a frame–wall building using five different effective stiffness models. It can be noted from the literature survey that most of the models or design code recommendations are based on the axial load ratio. However, some models consider the aspect ratio of the cross-section in addition to the reinforcement ratio. It can be seen from parametric studies [11,15] that the section dimensions and steel reinforcement are important factors that influence effective stiffness. Moreover, manual calculation of effective stiffness of the RC column is a laborious and time-consuming process. Therefore, the use of soft-computing techniques can be an alternative for estimating effective stiffness of RC columns. 

The predictive ability of genetic programming and gene expression programming (GP and GEP, respectively) models has attracted interest from researchers investigating structural engineering problems [16,17,18,19,20,21,22,23]. For example, Mansouri et al. [24] applied the GEP approach to improving the shear strength estimation of exterior RC beam–column connections. The compressive strength of recycled aggregate concrete for filling steel tube columns was predicted by Nour and Güneyisi [25] using the GEP approach. The axial compression strength of circular concrete-filled steel tube (CFT) columns was predicted in [26] using the GEP technique. 

It has been observed from the literature that the percentage of reinforcing steel, axial load, and section dimensions are the most influential parameters when evaluating the effective stiffness of columns. Most of the effective stiffness models reported in the literature do not consider all the above mentioned parameters together. In addition, manual computation of the effective stiffness of RC column sections is a very tedious process. To ease the tedious evaluation procedures, soft-computing models, such as GEP, can be used as an alternative to manual computations. GEP models are advantageous over other soft-computing techniques as they provide an explicit mathematical equation for prediction. In [9], model equations were presented based on the trained artificial neural network, but these are lengthy as the number of equations depends on the optimum number of neurons in the model. In GEP, single explicit equation is possible for a prediction model. It would be very useful for the design engineers as these can be used on the practical field to estimate the effective stiffness of RC columns without requiring any knowledge of soft-computing techniques. In the available literature, there is a dearth of simple mathematical models for estimating effective stiffness of RC columns considering percentage of reinforcing steel. Therefore, in this study, efforts have been made to develop effective stiffness formulations using GEP by considering the reinforcing steel percentage, axial load, and section dimensions. 

The paper has been further divided into five sections. Section 2 describes the concept of effective stiffness of RC sections based on strength. In Section 3, preparation of the database for the GEP model has been discussed. Section 4 presents the details of the modeling approach and application of the GEP model. In Section 5, the results obtained using the GEP model are discussed. Section 6 presents the conclusions of the present study.

## 2. Effective Stiffness of Reinforced Concrete Column Sections

From the moment–curvature relationship, the flexural stiffness of a reinforced concrete section can be expressed as follows:

(1) $$EI_{e}=\frac{M_{y}}{\phi_{y}}$$

where $I_{e}$ is the effective moment of inertia, E is the modulus of elasticity of concrete, $M_{y}$ is the yield moment capacity, and $\phi_{y}$ is the yield curvature of the section. 

It has been observed that for a given section, the yield curvature does not depend on variations in the moment capacity, axial load ratio, and reinforcement ratio of RC columns. However, for high material strength ($f_{c}$ > 50 MPa and $f_{y}$ > 600 MPa), this assumption does not hold good [1,27]. In the present study, the considered material strengths ($f_{c}$ = 30 MPa and $f_{y}$ = 500 MPa) were below the prescribed limit. Therefore, for a given section, a constant yield curvature value can be assigned. According to Priestley [1], the yield curvature values of RC rectangular columns can be obtained as Equation (2):

(2) $$\phi_{y}=2.10\;\frac{\epsilon_{y}}{h_{c}}$$

where $\epsilon_{y}$ is the yield strain of the reinforcing steel and $h_{c}$ is the column depth in the direction of the applied earthquake force. Yield moment capacity of column sections is to be obtained from the column interaction diagram for each column. Knowing the values of yield curvature and yield moment of the section, the effective stiffness ratio, r $\left(=EI_{e}/EI_{g}\right)$ can be evaluated with the help of Equation (1). Here, $I_{g}$ is the moment of inertia of the cross-section. 

## 3. Preparation of Database

**Figure 1:**
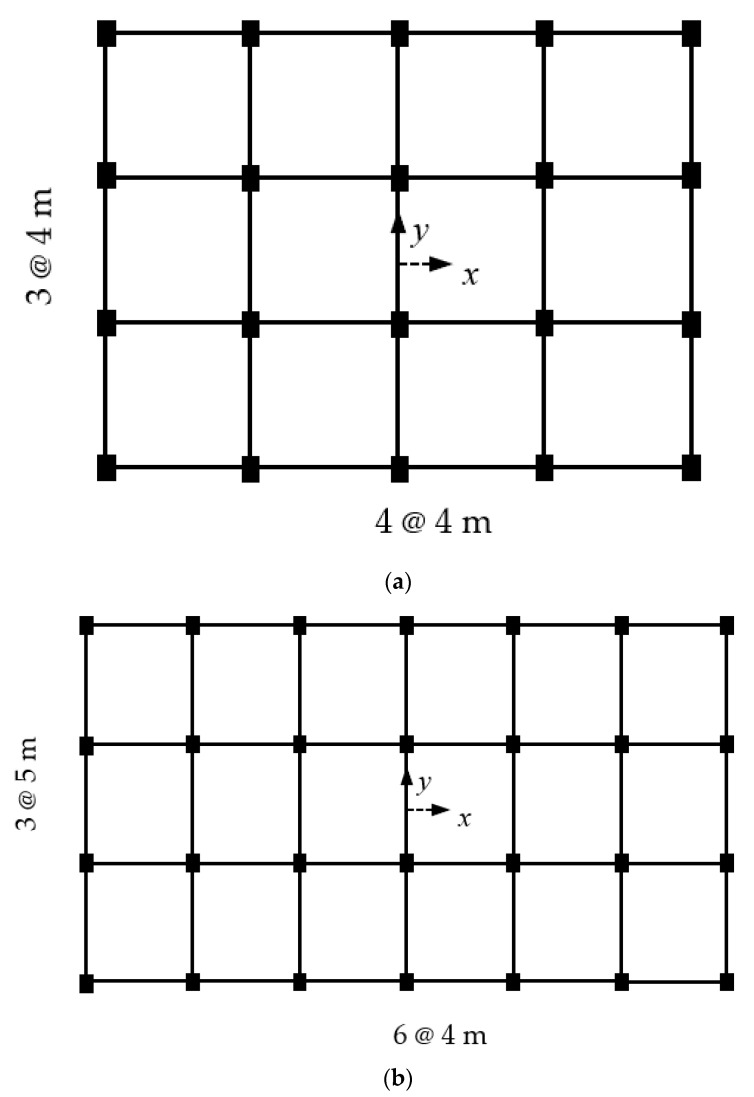
Building plans considered in the study (**a**) Plan I (**b**) Plan II.

For the present study, the database was collected from a previous study by the authors [9]. The database of effective stiffness ratios (*r*) of column sections was prepared from a set of buildings that had been designed via the unified performance-based design (UPBD) method [28]. RC frame buildings of two different plans, as shown in Figure 1, were considered with varying numbers of stories (5-, 8-, 10-, and 12-storey). Frame bays of plan I (Figure 1a) were spaced at 4 m in both the *x*- and *y*-directions. In plan II (Figure 1b), the bays were spaced at 4 m and 5 m in the *x*- and *y*-directions, respectively. The buildings were analyzed and designed for various performance levels (IO = immediate occupancy, LS = life safety, and CP= collapse prevention) using SAP2000 software. The database of 226 samples of effective stiffness ratios (r) of column sections was manually computed, as per the procedure defined in Section 2. To ensure the input parameters were spread over a large range, columns from all floors and locations were considered. In the present model, the longitudinal rebar percentage (*ρ_t_*), axial load (*P*), and depth of the column in both directions (*D_x_*, *D_y_*) are considered as input parameters to obtain the effective stiffness ratios of columns in both the orthogonal directions ($r_{x}\;\mathrm{and}\;r_{y}$) as output. The statistical parameters of the total dataset are listed in Table 1. 

## 4. Details of the Modeling Approach and Construction of the GEP Model

Ferreira [29] initially designed GEP, which is an innovative evolutionary algorithm [30]. In addition, GEP studies have an architecture established by two units: (1) an expression tree (ET) and (2) a chromosome. The solution considered in the ET is programmed within the chromosome. In this regard, this procedure of translating the chromosome in an ET is regarded as equivalent to biological genes programmed in DNA contained within proteins. Through GEP, the main limitations of the preceding genetic method (GA and GP) are eliminated, including the problems of using genetic operators over the trees (e.g., recurrent infeasible solution generation), code complexity/growth, and numerous syntactically unacceptable arrangements via genetic variation that destroy the computational origins [31]. In the GA, any mathematical expression is used as a fixed-length representative string (chromosome). According to Davis and Principe [32] and Cevik [33], a GA is characterized by nonlinear units with various shapes and sizes (parse trees) in the GP.

In contrast, the mathematical expression is translated by the GEP as simple, compact, linear, and relatively small fixed-length strings. In addition, genetic handling is easy via the expression of trees of various shapes and sizes. In the chromosome, GEP genetic operators are used; however, these operators are indirect for the considered solution (ET).

**Figure 2:**
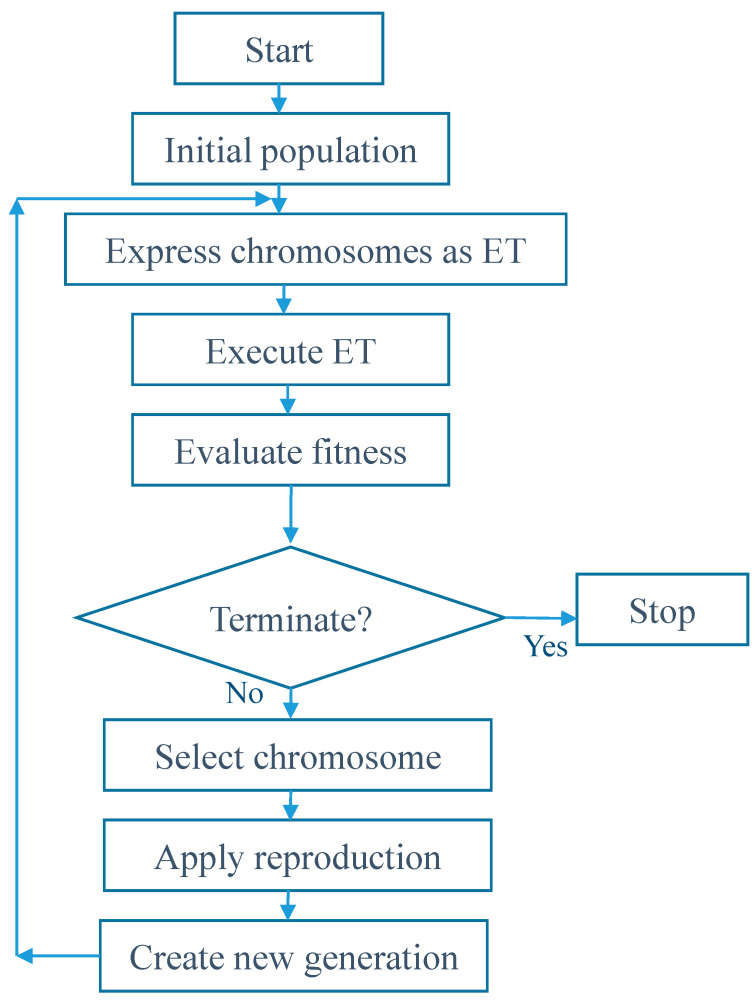
Flowchart of a Ferreira-based adjusted gene expression programming (GEP) algorithm.

Through this recreation technique, accompanied by the chromosome’s structure and its translation procedure in the expressing tree, unrestricted genetic modifications are always possible for creating effective expression trees. The very simplified form of genetic variation is one of the most important strengths of the GEP method because genetic operators act at the chromosome level. Some researchers believe that another merit of the GEP approach is related to its unique and multi-genetic features that lead to the development of more complicated programs consisting of numerous sub-plans. In this regard, the GEP approach with these features outperforms the GEP in terms of convergence speed for benchmark function results and organization issues [31]. The GEP involves five main elements: (1) function arrangement, (2) terminal arrangement, (3) fitness function, (4) control factors, and (5) stop circumstances. These components are treated via arithmetic performance, constants and variables, qualitative variables, an objective function, and numerical elements (e.g., population size, number of generations, and terminating criterion). To solve this problem, these elements should be specified using a GEP algorithm flowchart (Figure 2).

As shown in Figure 2, the algorithm was initiated with the unsystematic chromosome generation of the primary population. At that time, the fitness of each chromosome is assessed once the chromosomes are decoded into an ET and, subsequently, into a mathematical expression. Selecting the chromosomes is then based on the appropriateness of a duplicate with variation. The members of this novel generation are exposed to similar progressive practices: (1) genome expression, (2) choice environment confrontation, and (3) reproduction with alteration. This procedure is continued until a preferred quality is achieved in the solution for a definite number of generations. In GEP, the chromosomes are regularly chosen and duplicated into the next generation, regarding the fitness by roulette-wheel sampling with exclusiveness and thus assuring the cloning and survival of the best members to the next generation [31]. A summary of the GEP basics is presented in the next section. In this study, the form of fitness function is based on root mean square error (RMSE).

GEP plays an important role in linear chromosomes and ETs with a fixed length. The aim of an innovative language—for example, the Karva language for the GEP—is to improve the recitation and expression of any programmed and processed information in the chromosomes. The Karva language is an unequivocal and bilingual illustration system that can be regarded as a tree. In addition, the language involves a worldwide technique for efficiently demonstrating any scientific or common-sense notation that can be provided as a tree. In addition, Ferreira [29] believes that this global representation is linear, and could create new and accurate systems. The chromosomes represent mathematical expressions via the functions and terminals or constants and variables (for instance, a, b, c, and d) and involve one or more equal-length genes. Using a gene coding sequence, the ETs of GEP are concluded by the direct top to bottom and left to right readings of the ET. For example, a code is included in the expression that starts from “Q” (position 0) and terminates at “d” (position 7). In GEP, the coding sequences of these genes (Q × − + abcd) are known as K-expressions (from the Karva language), decoded to the ETs using the ET’s language. The ET can be understood after considering a gene’s sequence and vice versa. This indicates that a very complicated program characterized by its effective gene code can be selected with no loss in meaning. To properly interpret the gene’s coding sequence, the start location (the ET’s root) is located in the uppermost line under each function and is combined with numerous nodes of the branch (from left to right) as opinions exist regarding that function. The procedure was terminated as soon as a baseline consisting merely of terminals was shaped. GEP genes comprise a tail and head. The head (h) involves signs signifying both terminals and functions, whereas the terminals are only involved in the tail (t).

For each problem, the head’s length is selected and the tail length is assessed as a head function and the argument number (or maximum arity, *n*) using the equation t=h (*n*−1) +1. For example, a gene consisting of the function series (Q, ×, /, −, +) and the set of terminals (a, b) involves *n* = 2. If it is decided that h = 10, then t = 11; therefore, the gene’s length is 21. Hence, the gene’s code sequence terminates at position 10 whereas the end of the gene is at position 20. In cases where a mutation occurs via alterations to a terminal or function at any location in the coding sequence, various genes are prepared with a static length, probably coding for ETs with various shapes and sizes. For chromosomes with several genes, the considered solution is advanced by attaching the sub-ETs of the equivalent genes with the connecting functions (for example, +, −, ×, /, and, or, or, less than); therefore, the problem’s mathematical function is stated by the arrangement of sub-ETs. 

To select the head’s length and the number of genes in the chromosome structure, it is worthwhile to begin with single-gene short chromosomes. In this respect, h is gradually increased [29]. At the beginning of the analysis, the length of the head can be defined by the consumer regarding the sign quantities, such as preferred arithmetic functions and input variables. In addition, the GEP chromosome structure permits an actual functional phenotype/genotype association. Some alternations created in the GEP genome constantly lead to structurally accurate ET and syntactically precise plans. In fact, the diverse genetic operator set constantly improves the present genetic variety in GEP populations by creating effective ETs. 

The mutation is an accidental alternation of the coding sequence during chromosome selection. According to the rules, this mutation can occur wherever any sign in the chromosome can be changed into another (terminal or function) in the heads, whereas termini can merely turn into other termini in the tails. Instead, modifications through an inverse operator are limited to the gene heads. The rearrangement is driven on a chromosome section to another position in the chromosome. The transposing operator haphazardly duplicates the chromosome fragments and places them in another location. Recombination is an alteration process on a chromosome pair. Using the GEP’s recombining operator (or crossover), two parent chromosomes are selected and paired randomly to transact some features within them via three diverse operations (one-and two-point recombination and gene recombination). GEP operators are used with a definite likelihood of a chromosome (or operator rate) specified by the user prior to analysis. It is stated that the inversion and mutation rates are usually utilized within a range of 0.01–0.1. Furthermore, the recombination and transposition rates alternate in the range of 0.1–1, although moderate rates (0.1–0.4) have typically been proposed for them [30,31]. Alternatively, a characteristically universal crossover rate of 0.7 is utilized for a one-point recombination rate that relies on the additional operators’ rates [29]. 

According to Ferreira [29], the process of model derivation can be listed through the GEP via a series of steps:

(1) Arbitrarily dividing the dataset into testing and learning subsets;
(2) Collecting the general background (for example, population size, chromosome number, head length, linking function, and number of genes), rate of fitness function, and genetic operators for modeling the data;
(3) Selecting a suitable function set (mathematical functions and arithmetic operators);
(4) Administrating the evolutionary procedure until the achievement of the prediction results;
(5) Exhibiting the best-of-run model in ETs and changing the mathematical expression.

Appropriate operator rates and settings typically rely on the number of probable solutions and the difficulty of the problem. These constraints can be based on some formerly proposed values and numerous trials. The improvement of the GEP models is provided in detail in the next section.

In this work, the use of GEP is represented as a tool for predicting the effective stiffness ratio of columns in both directions (*r_x_* and *r_y_*), using a database obtained from [9,34]. An appropriate formula is obtained using the data bank of the computed column stiffness from the analyzed RC frame building sets of various plans and elevations. The terminal set includes four fundamental parameters of the effective stiffness ratio of columns, *r_x_,* and *r_y_*, as expressed in Equation (3):

*r_x_* and *r_y_* = *f* (*ρ_t_*, *P*, *D_x_*, *D_y_*) (3)

where *ρ_t_* is the percentage of longitudinal reinforcement (%), *P* is the axial load (×10^3^ kN), and *D_x_* and *D_y_* are the depth of the column section in each orthogonal direction (m). According to this database, the model was organized using four input variables within the GEP method. Based on these input variables within the GEP approach model, the effective stiffness ratio of the columns in both directions can be predicted. The findings of the model training and testing sets and the experimental results were compared. These results demonstrate that the GEP is a robust method for predicting *r_x_* and *r_y_*. The learning of functions is performed by the addition of arithmetic functions. The selected functions are given by the following equations:

(4) $$r_{x}=D_{y}\left(\frac{\rho_{t}+D_{y}}{D_{x}+5.25(D_{x}-(D_{y}-D_{x})\frac{P}{\rho_{t}})}\right)$$

(5) $$r_{y}=\left(\rho_{t}+P^{0.25}\right)\left(\frac{D_{x}-0.0077}{6.31D_{y}}\right)$$

To set variations in the database, a combination of genetic operators, including transposition, crossover, and mutation, was used. In this study, 180 out of 226 data sets (80%) were employed to train the GEP model and the remaining 46 (20%) datasets were used to test the GEP model.

## 5. Results and Discussion

### 5.1. Statistical Analysis of the Results

The performance statistics of the GEP-based formulations for the entire database are summarized in Table 2. Based on a logical hypothesis, if a model yields *R* > 0.8, a strong correlation exists between the predicted and measured values. Nevertheless, it should be noted that *R*^2^ = 1 is never an indication that the prediction of the dataset is perfect [35]. It is only an index in which both the predicted and experimental datasets can be linearly correlated. Thus, in this study, the assumptions and accuracy of the proposed models are not based only on *R*^2^ values. Therefore, the study adopted other proven statistical indices, including mean square error (MSE), mean absolute percentage error (MAPE), and root mean square error (RMSE), given in the form of Equations (6)–(8).

(6) $$\mathrm{MAPE}=\frac{1}{n}\overset{n}{\underset{i=1}{\sum }}\left|\frac{a_{i}-c_{i}}{a_{i}}\right|\times 100$$

(7) $$\mathrm{MSE}=\frac{\sum_{i=1}^{n}{\left(a_{i}-c_{i}\right)}^{2}}{n}$$

(8) $$\mathrm{RMSE}=\sqrt{\frac{\sum_{i=1}^{n}{\left(a_{i}-c_{i}\right)}^{2}}{n}}$$

where *a_i_* and *c_i_* are the actual and calculated outputs for the *i*-th output, respectively, and *n* is the number of samples. 

**Figure 3:**
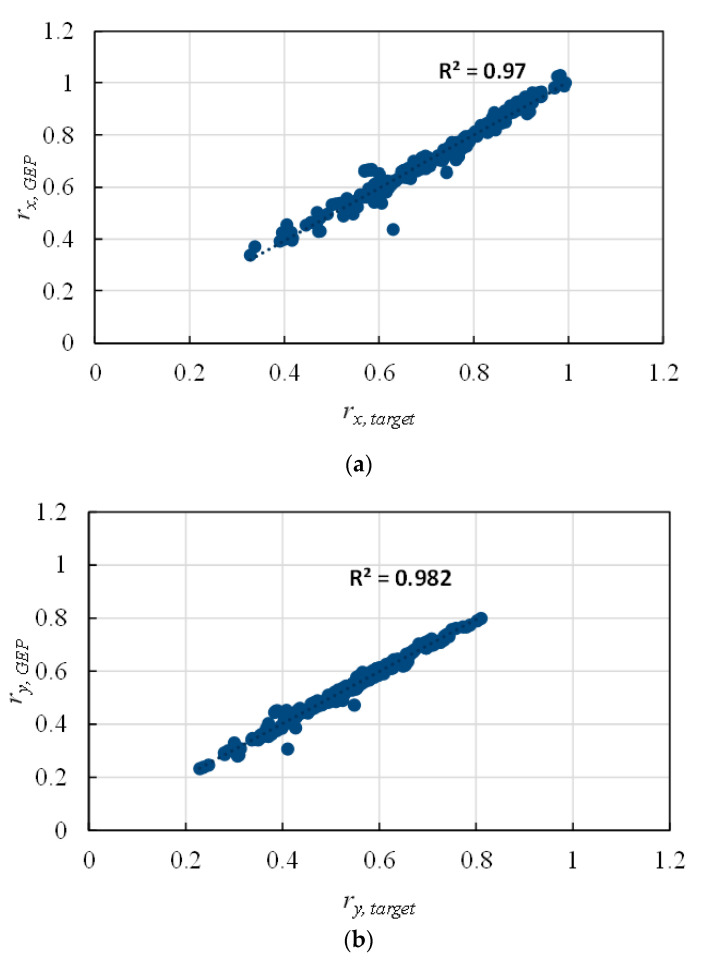
Predicted versus target effective stiffness ratio of reinforced concrete (RC) columns using the GEP model (**a**) *r_x_*, (**b**) *r_y_*.

As both training and testing data are involved in the model selection process (which is not a good practice), it is highly recommended to have three subsets in which the models are trained using training data (~70% of the data) and the model with the best performance on the testing data (~15%) is presented as the optimal model. Finally, this model was externally validated using unseen validation data (~15%). It can be inferred from Figure 3 and Table 2 that the GEP model predictions are accurate, with high *R* and low MAE and RMSE values. In Figure 3, the *x* axis shows the real values and the *y* axis indicates the predicted values by GEP.

### 5.2. Model Validity

To verify the GEP-based prediction model in this section, sensitivity and parametric analyses were performed. Using a sensitivity-like analysis affected by importance measures, it can be shown that GEP introduces features into evolved models that have little impact on a given model’s behavior. In addition, parametric analysis was used to study the response of the predicted outputs from the GEP equations to a set of predictor variables. The robustness of a design formulation is specified by evaluating the agreement between the predicted output values and the basic physical behavior of the investigated system [36].

#### 5.2.1. Sensitivity Analysis

Sensitivity analysis investigates the contribution of input parameters to the output predictions. For the sensitivity analysis, a simple procedure was proposed. The sensitivity percentage of the output to each input parameter can be determined using the following formulas [37]:

(9) $$N_{i}=f_{\max}\left(x_{i}\right)-f_{\min}\left(x_{i}\right)$$

(10) $$S_{i}=\frac{N_{i}}{\sum_{j=1}^{n}N_{j}}\times 100$$

where *f*_max_(*x_i_*) and *f*_min_(*x_i_*) are the maximum and minimum values, respectively, of the predicted output over the *i*-th input domain in which the other input values are equal to their mean values. The results of the sensitivity analysis for the estimation of the effective stiffness of RC columns are listed in Table 3. This table illustrates that the most influential input variable in the effective stiffness ratio of RC column sections in the *x*-direction is *D_x_*, followed by *D_y_*, *P*, and *ρ_t_*. The sensitivity analysis for RC column sections in the *y*-direction identified *D_y_*, *P*, *ρ_t_*, and *D_x_* as the most influential variables in the prediction of *r_y_*.

#### 5.2.2. Parametric Analysis

**Figure 4:**
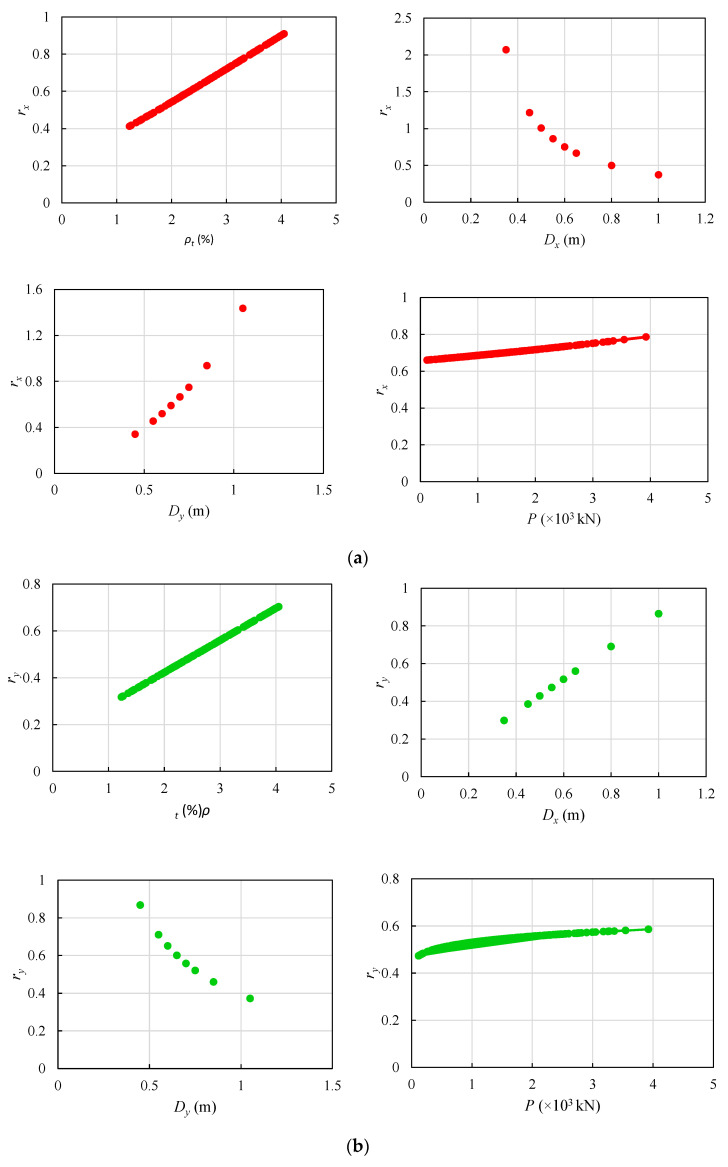
Effective stiffness ratio of RC column sections parametric analysis in the GEP-based model; (**a**) *r_x_* (**b**) *r_y_*.

For better validation of the GEP-based formulations, a parametric analysis was conducted following the procedure suggested in [36,37]. In general, the proposed models are physically meaningful. However, it is not always possible to obtain explicit models using artificial intelligence (AI) techniques. In such cases, the influence of each input variable on each output has to be studied to show that the proposed functions provide physically meaningful and not arbitrary trends. Figure 4 presents the tendency of the effective stiffness ratio predictions to the variations of the design variables, *ρ_t_*, *P*, *D_x_*, and *D_y_*.

As presented in Figure 4, *r_x_* continuously increases with increasing *ρ_t_*, *P*, and *D_y_*, which agrees with the trend reported by Kuntia and Ghosh [12] Similarly, *r_y_* increases with increasing *ρ_t_*, *P*, and *D_x_*. In contrast, *r_x_* and *r_y_* decrease with increasing *D_x_* and *D_y_*, respectively. This trend can be validated by the observations of Kumar and Singh [11].

## 6. Conclusions

An innovative model was established to predict the effective stiffness ratios of RC columns using the GEP method. A combination of genetic operators, including transposition, crossover, and mutation, was used in the study. The results indicate that the GEP model can successfully predict the effective stiffness ratios of the RC columns. GEP-based explicit formulations for predicting effective stiffness ratio were obtained and were compared with the generated database results. Various statistical evaluation criteria were used to evaluate the fitness accuracy of the model and the present equations. It was found that the suggested model displays the least errors (RMSE, MSE, and MAPE) and the highest correlation coefficients (*R*) of 0.983 and 0.990 for training and testing purposes, respectively. This establishes the prediction power of the GEPs. The validity of the model was established using sensitivity analysis and parametric analysis. The results of the parametric analysis being verified with that of similar studies reported in the available literature show that the model has captured all the input parameters correctly. It should be mentioned that the suggested model based on GEP in this study is provided in an obvious form and involves an extensive and varied range of input parameters. Hence, the suggested *r_x_* and *r_y_* can be calculated, allowing the use of the model in real-world situations.

## Figures and Tables

**Table 1:** Statistical parameters of the total data set.

Parameters	$r_{x}$		$r_{y}$		$\rho_{t}\;(\%)$		$D_{x}\;(m)$		$D_{y}\;(m)$		$\mathrm{Load}\;(\times 10^{3}\;\mathrm{kN})$	
	Min.	Max.	Min.	Max.	Min.	Max.	Min.	Max.	Min.	Max.	Min.	Max.
Range	0.328	0.994	0.229	0.81	1.23	4.05	0.35	1	0.45	1.05	0.116	3.922
Mean	0.709		0.531		2.87		0.63		0.72		1.388	
Standard Deviation	0.148		0.121		0.75		0.18		0.16		0.794	

**Table 2:** Performance results of the GEP model.

Model	*r_x_*_, Target_ vs. *r_x_*_, GEP_			*r_y_*_, Target_ vs. *r_y_*_, GEP_		
	*R* ^2^	RMSE	MAE	*R* ^2^	RMSE	MAE
Train	0.967	0.027	0.018	0.978	0.017	0.011
Test	0.980	0.027	0.020	0.991	0.015	0.010
Validation	0.972	0.024	0.018	0.986	0.013	0.010

**Table 3:** Sensitivity analysis of the input parameters in the gene expression programming (GEP) model.

Input	*ρ_t_*	*D_x_*	*D_y_*	*P*
Sensitivity (%) for *r_x_*	1.89	73.47	17.16	7.48
Sensitivity (%) for *r_y_*	10.66	0.78	55.41	33.14
